# Clinical and neuroimaging characteristics in mild-type infantile acute subdural hematoma: report of four cases

**DOI:** 10.1007/s00381-023-06089-7

**Published:** 2023-08-15

**Authors:** Nobuhiko Aoki

**Affiliations:** 1 Department of Neurosurgery, Bethlehem Garden Hospital, 3-14-72, Umesono, Kiyose-Shi Tokyo, 204-0024 Japan; 2 Department of Neurosurgery, Tokyo Metropolitan Tama Medical Center, 2-8-29, Musashidai, Fuchu-Shi Tokyo, 183-8524 Japan

**Keywords:** Abusive head trauma, Infantile acute subdural hematoma, Mild-type, Retinal hemorrhage, Shaken baby syndrome

## Abstract

**Purpose:**

Infantile acute subdural hematoma (IASDH) has a limited age distribution and mostly benign clinical features. Mild-type IASDH has a stereotypical clinical course which, however, has been described in only a few studies.

**Methods:**

Four male infants (aged 6–10 months; mean age: 7.5 months) were diagnosed as mild-type IASDH associated with retinal hemorrhage (RH) after suffering from occipital impact in a fall. The present case series reviews their clinical features and neuroimaging findings, including CT and MRI findings.

**Results:**

All the infants fell backwards from a standing or sitting position onto a soft surface, striking the occipital region. They began crying on impact and then soon afterwards exhibited seizure-like activity or recurrent vomiting. CT and MRI revealed a thin, unclotted subdural hematoma (SDH) without mass effect or brain parenchymal abnormality. Various degrees of bilateral RH were observed. On the day of symptom onset, all infants returned to baseline, and follow-up more than 5 years revealed normal development with no deficits.

**Conclusions:**

Mild-type IASDH with retinal hemorrhage presents with seizure-like activity or recurrent vomiting preceded by crying after an occipital impact on a soft surface. The clinical course of IASDH is followed by rapid recovery on the day of symptom onset. CT and MRI findings reveal a small, unclotted SDH without a mass effect or cerebral parenchymal abnormality.

## Introduction 

Infantile acute subdural hematoma (IASDH) has been reported since the 1960s in Japan [1]. However, because most cases were published in Japanese-language journals, coupled with the frequent criticism that the diagnosis was being used to conceal cases of child abuse, the concept of IASDH is not widely accepted in the English-speaking world [2].

Recent Japanese reports demonstrate that patients suffering from IASDH, particularly the mild form, can be distinguished from those with abusive head trauma (AHT) through multidisciplinary assessment, including evaluation by a child abuse pediatrician [3, 4].

Mild-type IASDH is the most common form, constituting more than half of IASDH cases, and is recognized as a benign clinical entity in Japan. No previous studies have examined the neuroimaging features characteristic of mild-type of IASDH. The present study therefore aimed to clarify the characteristics of IASDH, particularly the mild-type, to address this omission.

## Materials and methods

Between 2013 and 2022, 38 patients under the age of 2 years were referred to the Department of Neurosurgery at Bethlehem Garden Hospital with symptoms attributable to an acute subdural hematoma (SDH). The full clinical data on the patients, their CT (in some cases also MRI) findings, and interview records were analyzed. Fifteen of the patients received the diagnosis of IASDH, which was originally defined as an acute subdural hematoma in infants caused by minor head trauma without loss of consciousness or any associated cerebral contusion [5]. Patients thought to have AHT based on an evaluation by a multidisciplinary team, including a child abuse pediatrician, were excluded.

Mild-type IASDH was diagnosed in eight of the 15 infants based on their normal consciousness status, absence of motor disturbances, and the presence of vomiting and/or irritability on admission [5]. After excluding four patients who were not evaluated by MRI, the remaining four patients with long-term follow-up (> 5 years) were analyzed. Table 1 shows the details of their history.

**Table1:** Clinical and neuroimaging summary of four cases with mild-type IASDH

**Case No**	**Age (months), sex**	**Presenting history**	**Site of impact/surface**	**Presenting sign & symptom**	**Ophthalmological examination**	**CT findings on admission**	**MRI findings (days after onset)**	**Management**	**Outcome**	**Follow-up (years)**
1	8,M	Fall while trying to stand	Occiput/carpeted floor	Generalized tonic convulsion	Bilateral multilayered retinal hemorrhage	Thin SDH mixed density	T1 12 days thin film-like high-intensity SDH	Observation	Normal development	10
2	10, M	Fall while standing	Occiput/carpeted floor	Generalized tonic convulsion	Bilateral multilayered retinal hemorrhage	Thin SDH mixed density with sediment formation	FLAIR 3 days thin film-like high-intensity SDH	Observation	Normal development	9
3	7,M	Fall from sitting position	Occiput/cushion mattress	Flaccid posture, floppy	Bilateral multilayered retinal hemorrhage	Thin SDH high- &iso-density	FLAIR 19 days thin film-like high-intensity SDH	Observation	Normal development	6
4	6, M	Fall after trying to stand	Occiput/carpeted floor	Recurrent vomiting & irritability	Bilateral multilayered retinal hemorrhage	Thin SDH low density	T1 11 days thin, irregular shaped high-intensity SDH	Observation	Normal development	5

### Case 1

An 8-month-old, male patient with no significant medical history fell backward while trying to stand and struck his occipital area against a carpeted floor. The infant immediately began crying. While holding him in her arms, his mother noted signs of altered consciousness, seizure-like activities, including an upward gaze, and tremor of the left arm followed by generalized tonic convulsion. The infant was taken to a nearby emergency room.

**Fig. 1:**
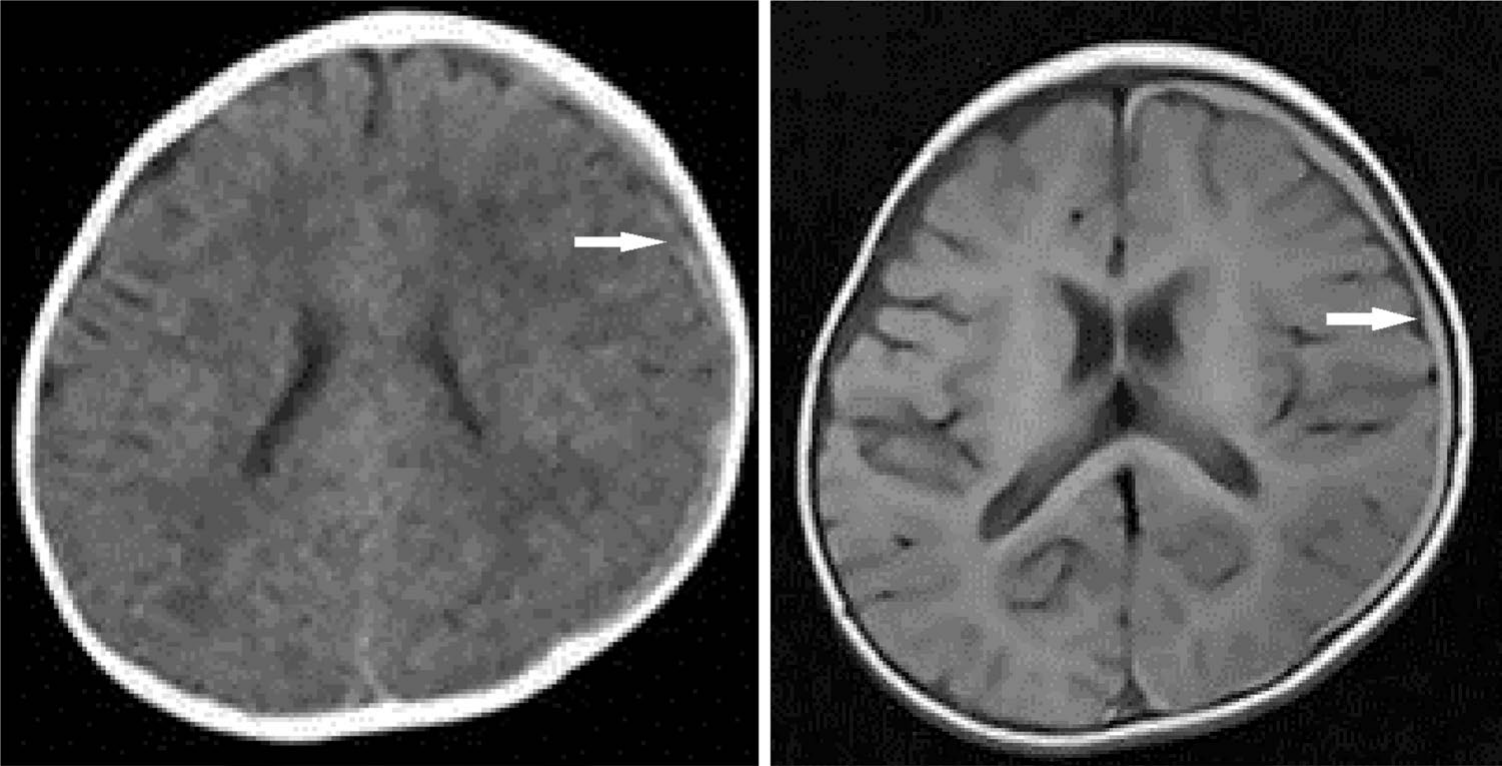
Case 1, male aged 8 months. Left: CT on arrival showing thin, irregularly shaped SDH, indicating an unclotted SDH on the left side (arrow). Right: MRI (FLAIR) 12 days after symptom onset demonstrating a thin, film-like, high-intensity SDH on the left convexity (arrow). No mass effect or parenchymal abnormality was observed on the other sequences (not shown)

**Fig. 2:**
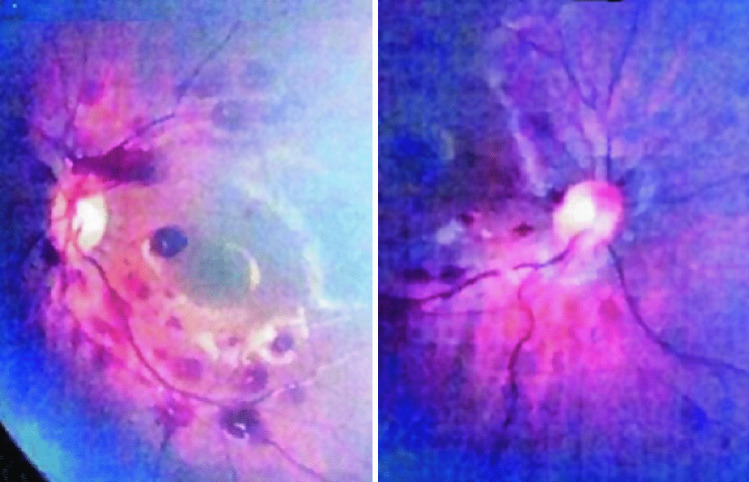
Case 1, fundoscopy on arrival demonstrating multiple, bilateral, multilayered retinal hemorrhages. Left: right eye. Right: left eye

When the physician began to examine the patient, he cried again and displayed purposeful movement in all his extremities. A standard, general physical examination revealed no abnormalities. Although the patient returned to baseline on the same day, emergency CT revealed SDH on the left side (Fig. 1), prompting an ophthalmological examination, which found bilateral multiple, multilayered retinal hemorrhages (RH) (Fig. 2). The infant was admitted for further observation and underwent MRI on hospital day 12, which revealed a thin, film-like, high-intensity SDH on the left cerebral convexity (Fig. 1).

No mass effect or parenchymal abnormality was observed. At his 10-year follow-up examination, the patient had normal development with no deficits.

### Case 2

**Fig. 3:**
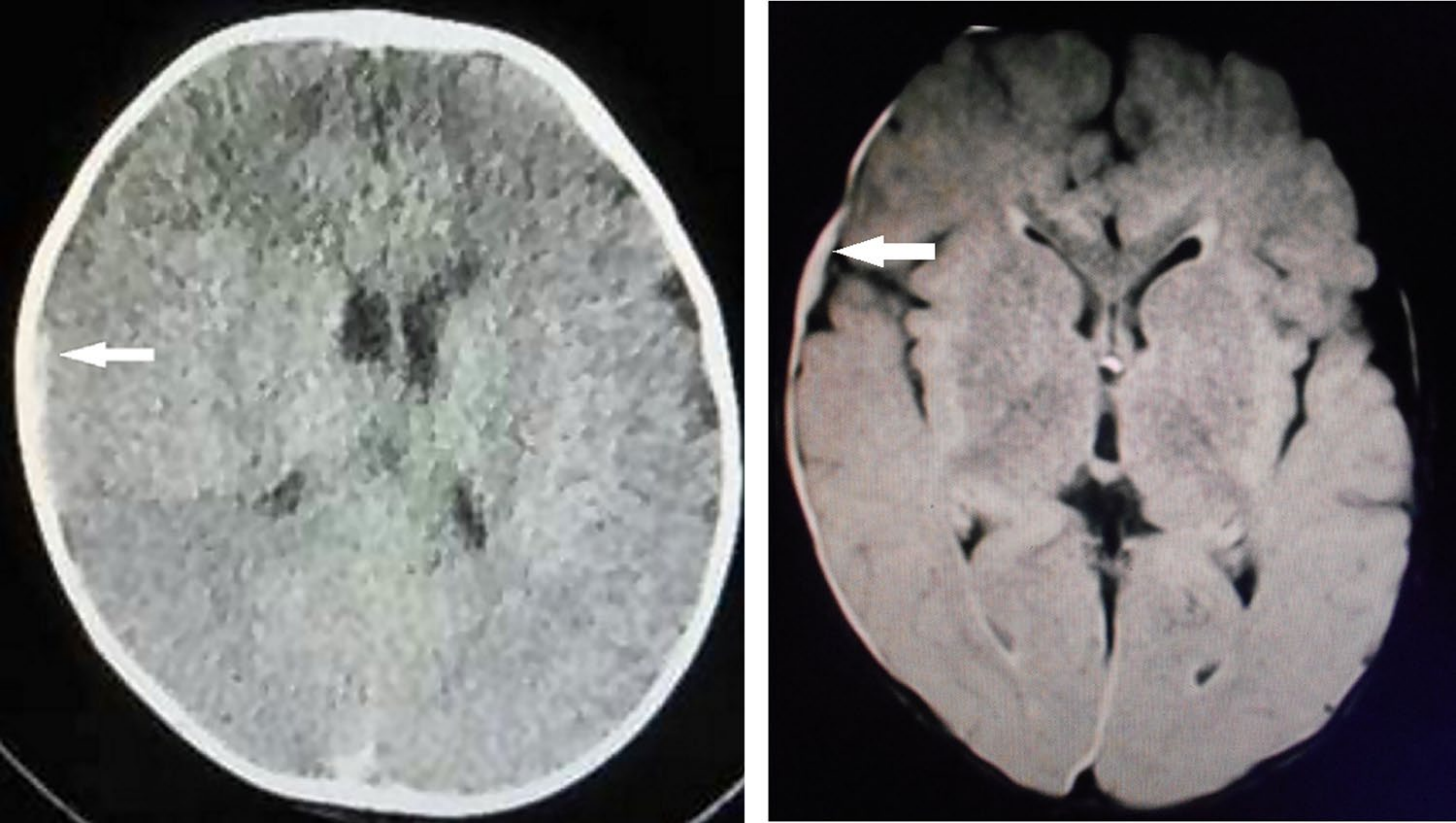
Case 2, male aged 10 months. Left: CT on arrival demonstrating a thin SDH with heterogeneous density on the right side (arrow). Note the low-density sediment formation in the upper part and high- density sediment formation in the lower part. Right: MRI (FLAIR) 3 days after symptom onset demonstrating a thin, film-like, high-intensity SDH on the right side (arrow). No mass effect or parenchymal abnormality was observed on the other sequences (not shown)

A 10-month-old, male infant who began walking fell and struck his occipital region on a floor covered by a 15-mm-thick carpet while trying to steady himself by grabbing a table leg. The scene was witnessed by his mother and grandparent members who were sitting in front of the patient at the time. The infant immediately began crying. While his mother held him in her arms to comfort him, he lost consciousness and exhibited seizure-like activity, including an upward gaze and tremor of the left upper extremities followed by a generalized tonic seizure. He was taken to an emergency room where he seemed alert and noted to return to baseline. Although no neurological abnormalities or external signs of trauma were observed, CT revealed thin SDH with mixed density on the right side (Fig. 3). Fundoscopy on the same day found bilateral multiple, multilayered RH. The infant was admitted for further observation for 10 days without presenting any neurological abnormalities. MRI on day 3 revealed a SDH on the left side and a posterior interhemispheric fissure. No mass effect or parenchymal abnormality was noted (Fig. 3).

During the observation at the hospital, the infant was asymptomatic. The subsequent outpatient follow-up for 9 years showed normal development.

### Case 3

**Fig. 4:**
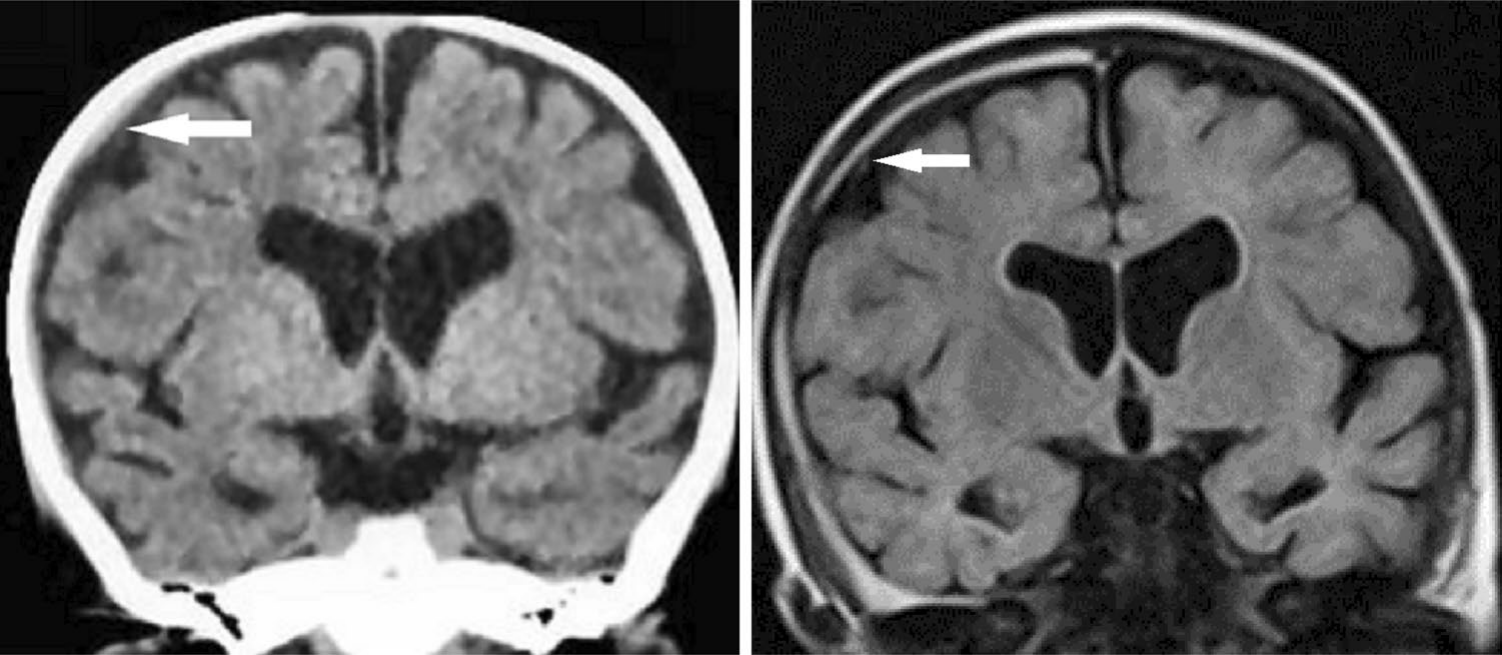
Case 3, male aged 7 months. Left: CT on arrival demonstrating tiny, high- and iso-dense SDH on the right cerebral convexity (arrow). Note the bilateral enlargement of the lateral ventricles and subarachnoid spaces. Right: MRI (FLAIR) 19 days after symptom onset demonstrating thin, film-like, high-intensity SDH on the right side (arrow). No mass effect or parenchymal abnormality was observed on the other sequences (not shown)

A 7-month-old, male infant with no significant medical history fell backwards from a seated position and struck his occipital region on a cushion mattress. The event was witnessed by his parents, who happened to be in front of the patient. The infant began crying immediately upon striking his head. His father held him in his arms and noted altered consciousness, upward deviation of both eyes, cyanosis, and flaccidity of the body. The infant was taken by ambulance to the emergency department. Enroute to the hospital, he regained consciousness and returned to baseline. In the emergency room, a pediatrician identified thin SDH with a maximum thickness of 2 mm on CT (Fig. 4) and decided to admit the patient for continued observation. No neurological abnormalities or external signs of trauma were noted. Two days after admission, fundoscopy revealed multiple multilayered RH on both sides. A full skeletal survey, including a 3-D cervical spinal CT, revealed no abnormalities. MRI performed 19 days after symptom onset revealed thin, film-like, high-intensity SDH on the right side (Fig. 4). No mass effect or parenchymal abnormality was observed. His clinical course was unremarkable, and the patient showed normal development at regular follow-up visits over 6 years.

### Case 4

**Fig. 5:**
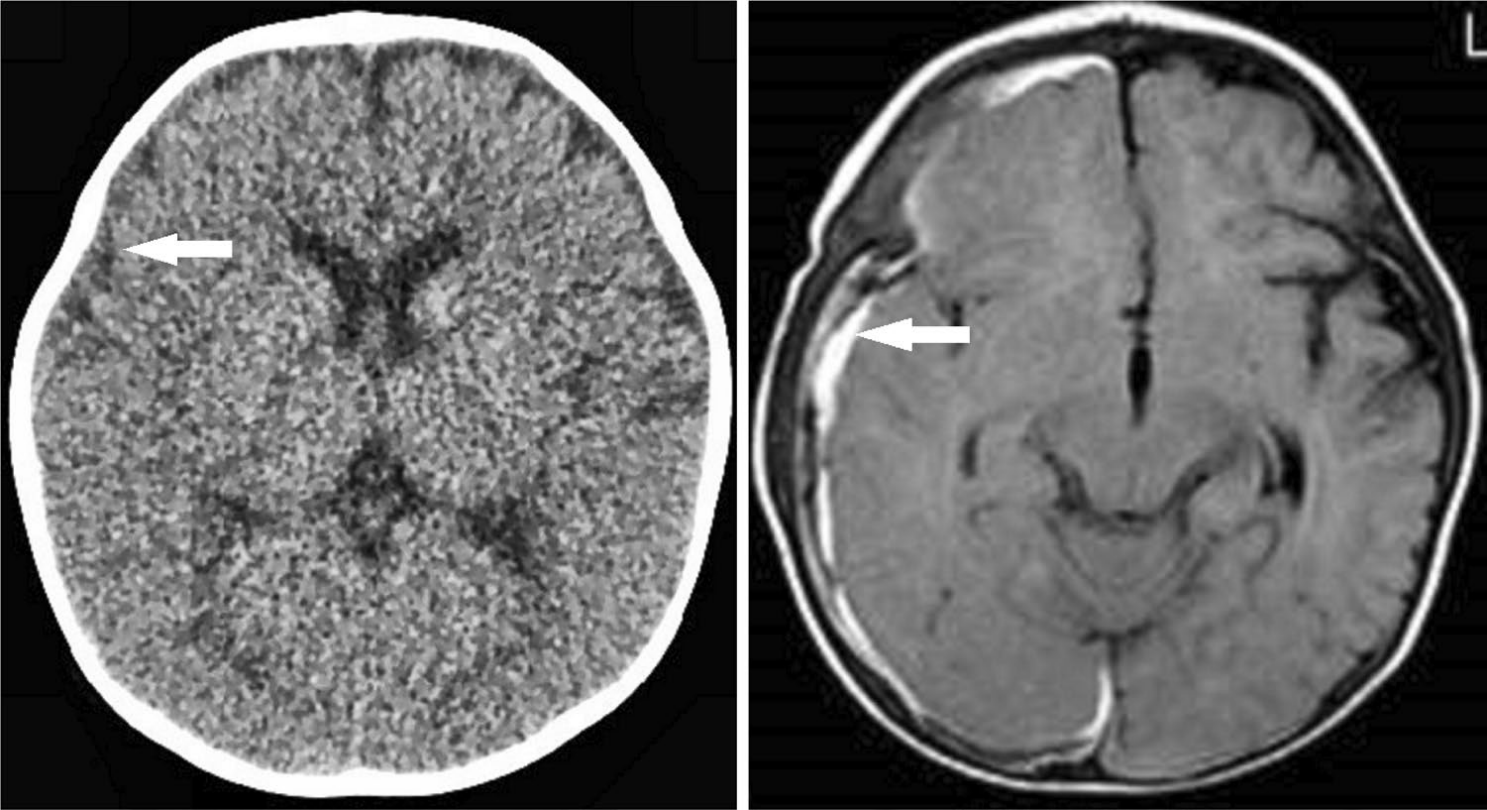
Case 4, male aged 6 months. Left: CT on arrival demonstrating thin, low-density SDH on the right side (arrow). Note that the density of the SDH is higher than that of the cerebrospinal fluid in the lateral ventricles and subarachnoid spaces on the left side. Right: MRI (T1-weighted image) 11 days after symptom onset demonstrating thin, high-intensity SDH on the right cerebral convexity (arrow). No mass effect or parenchymal abnormality was observed on the other sequences (not shown)

A 6-month-old, male infant with no significant medical history fell backward while trying to stand up in the living room and hit the occipital region on a carpeted floor. The infant began crying on impact and soon thereafter had vomiting incessantly. After signs of irritability appeared, the infant was taken to a nearby hospital where a physical examination failed to find any abnormality. However, the physician referred the infant to a pediatric hospital where CT was performed at the mother’s request, and an acute, low-density SDH on the left side was disclosed (Fig. 5). Although no neurological abnormality or external signs of trauma were noted, the infant was admitted for continued observation.

On the day of admission, the vomiting and irritability subsided, and the subsequent clinical course was uneventful. Two days after admission, fundoscopy revealed multiple, multilayered RH on both sides. MRI performed 11 days after symptom onset revealed thin, high-intensity SDH on the right side. No mass effect or parenchymal abnormality was noted (Fig. 5). The patient achieved normal developmental milestones over 5 years of regular follow-up visits.

## Discussion

IASDH has a limited age distribution, occurring chiefly between age 6 months and 10 months, when infants normally begin sitting or standing [3, 4]. As in previous, Japanese cases, all the infants in the present report immediately began crying on striking their head, indicating the presence of a lucid interval. Most patients with IASDH present a benign clinical course. Infants with mild-type IASDH typically return to baseline on the day of arrival at the emergency room, and follow-up visits demonstrate normal development. These clinical features of IASDH do not coincide with those of AHT, which has its peak incidence at ages 2 to 4 months and a generally poor prognosis [6–8].

Table 2 shows the difference between IASDH and AHT.

**Table2:** Comparison between accidental and nonaccidental infantile acute subdural hematoma (IASDH) without external signs of injury

	**Accidental IASDH**	**Nonaccidental IASDH**
Applied force	Minor head trauma	Abuse (high energy impact)
Main etiology	Disruption of bridging vein	Cerebral contusional tears
Primary brain injury	None	Common
Age distribution	Peak in 6 ~ 10 months	Widely distributed (including less than 3 months)
Gender	Marked preponderance in male	No preponderance
Recurrence	Rare	Not rare
Prognosis	Depending on volume of hematoma (mostly, benign clinical courses)	Poor
Retinal hemorrhage	Frequent	Common

(Cited from Ref. [1] with permission by the Society of Japanese Neurosurgery)

The most important pathology of the triad of SBS/SHT is encephalopathy. Encephalopathy in this setting represents a primary cerebral parenchymal injury, mostly brain contusion and diffuse axonal injury. As shown in Table 2 comparing nonaccidental head trauma to infantile acute subdural hematoma, primary brain injury is common in nonaccidental head trauma vs being absent in accidental IASDH. Now in Japan, the diagnosis of IASDH requires not only the details of the patient’s clinical profile but also confirmation of the absence of a primary cerebral parenchymal injury on MRI, particularly T2* and susceptibility-weighted images.

Neuroimaging findings of mild-type IASDH include a thin SDH with heterogeneous density, indicating the presence of a small, unclotted hematoma mixed with cerebrospinal fluid overlying a large surface area of the cerebral convexity. MRI is essential for confirming the absence of primary brain injury. However, infants with mild-type IASDH are not always evaluated by MRI. Since the patients become alert and asymptomatic on admission, their parents are reluctant to ask for MRI, which requires sedation.

No surgical intervention or autopsy studies have been done in infants with mild-type IASDH, and so the mechanism of injury involved in this condition is a matter of speculation.

Zouros et al. proposed that during infancy, tearing of the loosely adherent arachnoid envelope at the main arachnoid granulation site along the superior sagittal sinus may result in a considerable amount of CSF mixing with acute blood in the subdural space [9]. In the present series, the CT and MRI findings demonstrating thin, unclotted, widely diffused SDH in the cerebral convexity may be explained by cleavages in the dural border cell layer [10]. In this context, a traumatic rupture in the arachnoid granulation connecting columns of arachnoid cells to the venous sinuses might explain the mechanism inducing mild-type IASDH. Tearing of the bridging veins, which commonly causes symptomatic SDH with mass effect, could be followed by progressive hemorrhage through the cleavages in the dural border cell layer.

The clinical severity of IASDH depends on the volume of the subdural hematoma; thus, surgery is not indicated for mild-type IASDH. However, as seen in cases 2 and 4 in the present series, tiny or low-density SDH on CT might be overlooked, potentially resulting in the development of subacute or chronic SDH [11].

Now in Japan, it has become a duty for healthcare workers to report cases to childcare centers if there is any suspicion of child abuse. Four cases in this series were also reported to a childcare center on the suspicion of AHT [4].

During hospitalization, for the purpose of close observation, all four cases were investigated according to the recommended format, including information from precise history taking, family composition, and contacts in the victim records, to create a flow chart. As a result of this process, the diagnosis of AHT was excluded.

It is important to understand how common accidental IASDH relative to SDH associated with nonaccidental trauma in recent Japan. Akutsu N et al. described that unlike studies in other countries, more than half the infantile subdural hematoma cases were determined to be accidental. This suggests that the likelihood of an accidental subdural hematoma in infants varying by ethnicity. On the other hand, patients younger than 5 months, those with retinal hemorrhage, and those with seizures were found more likely to have suffered abuse, as in other countries. The diagnosis of abuse should not be made by simply applying the standards of one particular region to other regions; it is necessary to consider the possibility that there are cultural and racial differences unique to each region [3].

Shimoji K and his colleges described that as in other countries, AHT is a major condition which pediatric neurosurgeons encounter in Japan. The mechanisms of injury and the perpetrators of AHT seem to differ slightly between western countries and Japan. Additionally, it is the accidental infantile acute subdural hematomas that are chiefly reported in Japan. Therefore, great care and fair judgment are necessary when investigating child abuse in Japan [4].

Moreover, the incidence of accidental IASDH relative to the number of patients with SDH seen in nonaccidental injury was reported. Narisawa et al.’s recent, multicenter, retrospective study reviewed the clinical records of children younger than 4 years with head trauma who visited the study centers between January 2014 and August 2020. Of the 84 patients with SDH, 51 (60.7%) were judged to have received nonaccidental injury according to established criteria, including patients who were taken into temporary custody by the Child Guidance Center. On the other hand, of 30 falls from a height < 2 m (10 of 15 self-inflicted falls, 4 of 5 falls from a bed or sofa, 1 of 5 falls while being held by a parent, and no cases of being dropped by a caregiver), 20 were judged to have suffered accidental injury by the Child Guidance Center [12].

## Conclusions

Infants with mild-type IASDH present with crying followed by seizure-like activity or vomiting/irritability after striking the occipital region on a soft surface. On arrival at the emergency room, they are asymptomatic, presenting only retinal hemorrhage. CT and MRI findings are characterized by the presence of a thin, unclotted SDH without a mass effect or any cerebral parenchymal abnormality. CT findings may be overlooked if the physician is not aware of the possibility of the unique association of this type of SDH.

The basic differences in the SDH with RH associated with AHT in the English-speaking world and with IASDH in Japan may reside in the absence or presence of a primary cerebral parenchymal injury. Further studies are needed to elucidate the mechanism and predisposition of infants with IASDH.

The present study has several limitations. The population of Japan is racially homogeneous. In the present series, the patients were all Japanese infants who were referred to the study center for a second opinion. The small number of cases were all of mild-type IASDH only, thus potentially introducing a selection bias.

Finally, the comment by Dr. Anthony J. Raimondi on the author’s article concerning AHT in Japan is worth highlighting in the present controversy.

Anthony J. Raimondi, M.D. *Chicago, Illinois.*

“The conclusions of these authors are sound. By review of Japanese cases and those published in the American literature, they seriously question the tenet that coexisting subdural hematoma and retinal hemorrhages are pathognomonic of battery. Of course, they are right; one cannot ascertain that a head injury is caused by battery simply because subdural hematoma and retinal hemorrhage are both present. The point that the authors really should be making is that there is no way clinically to identify unequivocally the battered child. This is true in the United States and, I suspect, may very well be true in Japan” [13].

## Data Availability

This article does not include any data or material to be provided.
